# Evaluation of vitamin D receptor gene polymorphisms (ApaI and TaqI) as risk factors of vitiligo and predictors of response to narrowband UVB phototherapy

**DOI:** 10.1007/s00403-022-02348-w

**Published:** 2022-03-23

**Authors:** Youssef Elbayoumy Youssef, Heba Elsayed Abdelmoneim Eldegla, Rana Samir Mahmoud Elmekkawy, Mohammad Ali Gaballah

**Affiliations:** 1 Department of Dermatology, Andrology and STDs, Faculty of Medicine, Mansoura University, El-Gomhoria St., Mansoura, Egypt; 2 Department of Medical Microbiology and Immunology, Faculty of Medicine, Mansoura University, Mansoura, Egypt; 3 Mansoura University Hospital, Mansoura, Egypt

**Keywords:** Vitamin D receptor, ApaI, TaqI, UVB, Vitiligo

## Abstract

Vitiligo is acquired depigmentation due to multiple factors. Vitamin D in skin, through its receptors (VDR), regulates cell growth, differentiation, immune response and exerts both stimulatory and protective effects on melanocytes. The gene sequence encoding VDR has polymorphic forms such as ApaI and TaqI that may affect vitamin D actions. Narrowband ultraviolet B (NB-UVB) phototherapy became the mainstay of vitiligo treatment because of its efficacy and little side effects. The current work aimed at evaluating the possible association between VDR gene polymorphisms (TaqI and ApaI) and susceptibility of vitiligo and if they could be predictors of response to NB-UVB phototherapy in Egyptian vitiligo patients. 100 vitiligo patients indicated for NB-UVB phototherapy and 100 healthy age and sex matched controls were included. All participants were subjected to history taking, general and dermatological examinations, and VDR ApaI and TaqI gene polymorphisms analysis by PCR–RFLP. The patients received NB-UVB 3times per week for 6 months then revaluated. There was significant increase in Aa genotype of ApaI polymorphism in patients associated with significant increase in vitiligo activity. 66% of patient showed variable degrees of response to NB-UVB. The responders significantly had AA genotype of ApaI polymorphism. TaqI polymorphism showed nonsignificant effects on vitiligo susceptibility and response to NB-UVB. A allele of ApaI was significant independent predictor of NB-UVB phototherapy responders. VDR gene polymorphism (ApaI) may share in vitiligo pathogenesis and response to NB-UVB. Knowing the genetic background of the patient helps individualization of treatment to get better results.

## Introduction

Vitiligo is acquired chronic depigmenting disorder with prevalence 0.5–2% worldwide [1]. Clinically, it is characterized by well defined milky white macules and patches resulting from loss of epidermal melanocytes. The interplay of multiple suggested factors as genetic, neural, psychogenic, oxidative stress, biochemicals, minerals, and autoimmunity may induce vitiligo [2].

Vitamin D is a lipophilic hormone, its main source is the endogenous synthesis in the skin by ultraviolet B (UVB) radiation, and exerts its effects through the nuclear vitamin D receptor (VDR). The VDR gene is located on chromosome 12q12–q14 [3]. Vitamin D has various biological functions in the skin, such as immunomodulation and regulation of cell growth and differentiation. In addition, it exerts both stimulatory and protective effects on melanocytes. It increases melanogenesis and tyrosinase content of cultured human melanocytes by its antiapoptotic effect. In addition, vitamin D induces the immature melanocytes in the bulge region of hair follicles to produce melanin by stimulating their differentiation and expression of endothelin B receptor [4, 5].

Decreased serum 25(OH) vitamin D level showed significant positive association with the incidence of vitiligo [6]. Several VDR gene polymorphisms were studied in vitiligo such as ApaI (VDR 7975232 C > T), BsmI (VDR1544410 A > G), FokI (VDR 2228570 C > T), and TaqI (VDR 731236 T > C). These polymorphic forms of VDR may be associated with decreased VDR activity, reduced vitamin D actions, vitamin D deficiency and increased risk for development of vitiligo [7].

Narrowband UVB light (310–313 nm) (NB-UVB) phototherapy is effective widely used modality in treatment of vitiligo [8]. NB-UVB phototherapy is associated with improved cutaneous VDR expression, increased vitamin D synthesis and increased serum vitamin D level which may be one of its mechanisms of repigmentation [9–12].

The current work aimed at evaluating the possible association between VDR gene polymorphisms (TaqI and ApaI) and susceptibility of vitiligo and if they could be predictors of response to NB-UVB phototherapy in Egyptian vitiligo patients.

## Patients and methods

### Study design

This study included 100 vitiligo patients indicated for NB-UVB (310–313nm) phototherapy and 100 apparently healthy age and sex matched volunteers who do not have any medical condition (e.g., auto immune or inflammatory disorders) as controls. Patients were recruited from the outpatient clinics of Dermatology, Andrology and STDs department, Mansoura University Hospital and Mansoura Dermatology and Leprosy Hospital. All patients were already diagnosed as vitiligo based on clinical assessment, Wood’s light examination, dermoscopic evaluation and skin biopsy when indicated. None of the controls had any current evidence or family history of vitiligo, dermatologic diseases or autoimmune diseases. Informed written consents were obtained from all participants. This study was approved by the Institutional Research Board (IRB) of Faculty of Medicine, Mansoura University.

All participants were subjected to full history taking, thorough general assessment and complete dermatological examination including skin, hair, nails and oral cavity.

Vitiligo was classified clinically, based on the distribution and extension of lesions according to Nordlund [13, 14]:

1. Localized:
  - Focal: One or more macules with casual distribution.
  - Unilateral: One or more macules are localized in a unilateral body region, with a dermatomeric distribution: a typical feature is an abrupt stop of the lesions at the midline.
  - Mucosal: Unique involvement of mucous membranes.
2. Generalized:
  - Vulgaris: Presence of scattered stains extensively disseminated.
  - Acrofacial: Patches are localized on distal extremities and face.
  - Mixed: Coexistence of acrofacial and vulgaris forms.
3. Universalis: Depigmented lesions completely or almost completely cover the skin surface. Assessment of vitiligo extent and percentage of body involvement was done utilizing the Vitiligo Area Scoring Index (VASI) [15], while assessment of vitiligo activity was done utilizing the Vitiligo disease activity (VIDA) score [16]. A low VIDA score indicates less vitiligo activity. All patients were assessed by VASI score before start phototherapy and after having 72 sessions of treatment in 6 months (3 sessions per week) to evaluate the response of patients to phototherapy and to classify patients into responder and non-responder according to Bae et al. [17] who suggested that at least 6 months of treatment is required to determine the responsiveness to NB-UVB phototherapy. In addition, according to the observations of Vitiligo Working Group committee members, a slower than usual response could be seen in some patients, thus a minimum of 72 NB‑UVB sessions should be administered before labeling a patient as a non-responder [8].

The degree of repigmentation was evaluated based on all lesions in each participant. Patients showed any decrease of VASI score was considered responder. Response rate (percentage) was calculated as the number of participants who showed the corresponding repigmentation divided by the number of all patients enrolled in the study.

### Laboratory investigations

Three mL of venous blood were collected from patients on EDTA by venipuncture. The collected blood samples were stored at −20 °C until the time of genetic studies that was done in the genetic unit in Medical Microbiology and Immunology Department, Mansoura faculty of medicine.

DNA extraction from blood samples was done by using DNA extraction Kit (Fermentas, Germany). Isolated and purified DNA was extracted from whole blood through the use of proteinase K. Then, DNA was amplified by polymerase chain reaction (PCR) using Thermo Scientific DreamTaq Green PCR Master Mix (2X) # K1081 [18]. PCR Master Mix (2x) was a ready-to-use solution and primers were provided by (Sigma, UK). Primers for VDR genes were supplied in a lyophilized form and prepared according to manufacturer's instructions. The sequences of primer used are F-9A 5′CAGAGCATCGACAGGGAGCAA3′ and R-9A 5′CACTTCGAGCACAAGGGGCGTTAG 3′.

Then Agrose gel electrophoresis was done. Thermo Scientific GeneRuler 100bp plus DNA Ladder marker (catalog# SM0323) was run in one of the gel wells to determine the size of the amplified PCR product. The lid of the gel tank was closed and the electrical leads were attached so that the DNA would migrate towards the anode (red lead). When the bromophenol blue dye had reached 2/3 of the gel length, the power supply was turned off and the running tray was removed. The gel was photographed using a polarized camera and a UV transilluminator [19].

Restriction fragment length polymorphism (RFLP) was done using fast digest supplied by Thermo Scientific Fast Digest ApaI (#FD0674) lot: 00170641 and TaqI (#FD 1414) lot: 00164225. The PCR products were incubated with specific restriction endonucleases in heat block at 37 °C for 16 h when using fast digest ApaI and at 65 °C for 3 h when using fast digest TaqI. 10 μl of each reaction was loaded on 3% agrose gel to visualize separated bands by UV illuminator and identify the restriction sites [20].

### Statistical analysis

Data were entered and statistically analyzed using the Statistical package for social sciences (SPSS) version 21 (SPSS, Inc., Chicago, IL, USA). The normality of data was first tested with one-sample Kolmogorov–Smirnov test. Qualitative data were described using number and percent. Association between categorical variables was tested using Chi-square test, while Fischer exact test was used when expected cell count less than 5. Continuous variables were presented as mean ± SD (standard deviation) for parametric data and median for non-parametric data. The two groups were compared with Student^’^s *t* test for parametric data and Mann–Whitney test for non-parametric, while paired groups compared by Wilcoxon signed rank test. ANOVA test was used to compare more than two means, while Kruskal–Wallis test was used to compare more than two medians. Significant variables on univariate analysis of the predictors of cases responder entered into Logistic regression model using the enter statistical technique to predict the most significant determinants and to control for possible interactions and confounding effects. For all above mentioned statistical tests done, the threshold of significance is fixed at 5% level (*p* value). The results was considered significant when the probability of error is less than 5% (*p*  < 0.05).

## Results

All the patients completed the study till its end. There were no significant differences between patients and controls regarding age, sex, marital status, occupation and residence (Table 1). 20 patients were cigarette smokers, 12 patients were diabetics, 12 patients were hypertensive, and four patients had autoimmune thyroiditis. History of psychiatric trauma was present in 50 patients and family history of vitiligo was positive in 24 patients. Leukotrichia was detected in four patients. Course of the disease was stable in 60 patients and progressive in 40 patients with VIDA score ranged from -1 to 4 (median score 2). Duration of vitiligo ranged from 1 to 34 years (median 5 years). Type of vitiligo differed among patients as there were 42 patients with vitiligo vulgaris, 20 patients with mixed vitiligo, 18 patients with acrofacial vitiligo, 16 patients with focal vitiligo and 4 patients with unilateral vitiligo.

**Table 1:** Demographic data and vitamin D receptor genotypes and alleles of the studied groups

Variables	Patients group (*n* = 100)		Control group (*n* = 100)		Test of significance	*p*-value
Age (years)						
Mean ± SD Range	29.48 ± 13.15 7.00–57.00		33.24 ± 11.40 18.00–60.00		*t* = 1.72	0.087
Sex Male Female	*n*	%	*n*	%	χ^2^ = 2.06	0.151
	36 64	36.0 64.0	46 54	46.0 54.0		
Marital status						
Married Not married	58 42	58.0 42.0	64 36	64.0 36.0	χ^2^ = 0.757	0.384
Occupation						
Working Not working	42 58	42.0 58.0	48 52	48.0 52.0	χ^2^ = 0.727	0.394
Residence						
Urban Rural	44 56	44.0 56.0	54 46	54.0 46.0	χ^2^ = 2.01	0.157
Taq I						
Homo TT Homo tt Hetero Tt	50 14 36	50.0 14.0 36.0	46 8 46	46.0 8.0 46.0	χ^2^ = 3.02	0.221
Apa I						
Homo AA Homo aa Hetero Aa	48 4 48	48.0 4.0 48.0	66 14 20	66.0 14.0 20.0	χ^2^ = 19.9	< 0.001*
Taq I alleles						
T t	136 64	68.0 32.0	138 62	69.0 31.0	χ^2^ = 0.046	0.830
Apa I alleles						
A a	144 56	72.0 28.0	152 48	76.0 24.0	χ^2^ = 0.832	0.362

*t* Student^’^s *t* test, χ^2^ chi-square test

^*^P is significant if < 0.05

Regarding ApaI genotype, Hetero Aa was significantly higher in patients group than control group. Homo AA and Homo aa were significantly higher in control group than patients group. No significant differences were found between patients and controls regarding TaqI genotype, TaqI alleles and ApaI alleles (Table 1). The frequency distribution of combination of VDR genotypes for ApaI and TaqI polymorphic sites in patients showed that AaTT genotype was significantly the most frequent (32 patients) followed by AATt genotype (24 patients) and AATT genotype (16 patients) (*p* = 0.003).

In general, no significant associations were detected between the genotype and alleles distribution of the patients and age, sex, special habits, course of vitiligo (progressive or stable), associated co-morbidities, history of psychiatric trauma, family history of vitiligo, presence of leukotrichia, type of vitiligo, VIDA score, VASI score before treatment, VASI score after treatment and percent change of VASI score. However, there were exceptions as patients with Homo TT showed significant association with mixed type of vitiligo (16 patients) (*p*=0.008). In addition, patients with Homo aa genotype (*n*=4) (all of them were non-responders to NB-UVB) significantly showed the highest VIDA score compared with other ApaI genotypes (*p*=0.028) with median score 3.5 followed by Aa (median score 2) and lastly AA (median score 1.5).

The overall median VASI score for all patients (*n* = 100) showed significant decrease after 6 months of NB-UVB treatment as it was 2.50 (0.25–8.75) before treatment and became 1.92 (0.10–6.75) (*p* < 0.001). The response rate was 66% (66 responders and 34 non-responders).

Regarding the relation between treatment response and VDR genotypes and alleles, Homo AA genotype was significantly higher in responders, while Homo aa genotype was significantly higher in non-responders. In addition, the response was significantly higher in patients carrying A allele vs. patients carrying a allele. On the other hand, TaqI geonotypes and alleles distributions nonsignigicantly affect the response to NB-UVB therapy (Table 2).

**Table 2:** Relation between response and different criteria of the patients

Variables	Responders (*n* = 66)		Non responders (*n* = 34)		χ^2^	*p*-value
	No	%	No	%		
Residence						
Urban Rural	34 32	51.5 48.5	10 24	29.4 70.6	4.45	0.035*
Type of vitiligo						
Focal	6	9.1	10	29.4	6.89	0.009*
Unilateral	2	3.0	2	5.9	FET	0.603
Acrofacial	16	24.2	2	5.9	2.93	0.086
Vulgaris	24	36.4	18	52.9	2.53	0.112
Mixed	18	27.3	2	5.9	6.41	0.011*
Genotype						
Taq I Homo TT Homo tt Hetero Tt	36 8 22	54.5 12.1 33.3	14 6 14	41.2 17.6 41.2	1.67	0.433
Apa I Homo AA Homo aa Hetero Aa	36 0 30	54.5 0 45.5	12 4 18	35.3 7.7 52.9	9.75	0.008*
Taq I alleles						
T t	94 38	71.2 28.8	42 26	61.8 38.2	1.84	0.175
Apa I alleles						
A a	102 30	77.3 22.7	42 26	61.8 38.2	5.35	0.021*
VIDA score (median & range)	1.0 (−1.00–4.00)		2.00 (0.00–4.00)		*Z* = 2.16	0.009*
VASI score before treatment (median & range)	3.00 (0.50–8.75)		2.25 (0.25–3.90)		*Z* = 2.82	0.005*
VASI score after treatment (median & range)	1.75 (0.10–6.75)		2.25 (0.25–3.95)		*Z* = 1.66	0.097
% of change in VASI score (median & range)	−45 (−91.30/4.35)		0 (−10/12.86)		*Z* = 7.51	< 0.001*

*FET* Fisher's exact test; *Z* Mann–Whitney test; χ^2^ chi-square test; *t* Student’s t test

^*^P is significant if < 0.05

In addition, responders were significantly from urban areas and they significantly had mixed type of vitiligo. On the other hand, focal type of vitiligo was significantly more in the non-responders. VIDA score was significantly lower in responders. VASI score before treatment was significantly higher in responders vs. non-responders, but VASI score after treatment showed no significant difference between the two groups. The percent change in VASI score with treatment showed significant decrease in responder group with median change -45% (Table 2). Finally, there were nonsignificant differences between responders and non-responders regarding age, sex, marital status, occupation, special habits, duration of vitiligo, course of vitiligo (stable or progressive), associated co-morbidities, history of psychiatric trauma, family history of vitiligo and presence of leukotrichia.

After logistic regression analysis, the significant independent predictors of responder in patient group were mixed type of vitiligo (OR = 6), absence of focal vitiligo (OR = 4.16), urban residence (OR = 2.55), ApaI A allele (OR = 2.11), high VASI score before treatment (OR = 1.63), high percent of change in VASI score with treatment (OR = 1.3) and low VIDA score (OR = 0.69) (Table 3).

**Table 3:** Logistic regression analysis of independent predictors of responders in patient group

Independent predictors	*B*	*p* value	OR (95% CI)
Residence			
Urban Rural (r)	0.936	0.037*	2.55 (1.06–6.15)
VIDA score	−0.376	0.01*	0.69 (0.51–0.91)
Focal vitiligo			
Yes (r) No	1.427	0.012*	4.16 (1.36–12.7)
Mixed vitiligo			
Yes No (r)	1.792	0.022*	6.0 (1.3–27.6)
VASI score before treatment	0.491	0.004*	1.63 (1.17–2.27)
% of change in VASI score	0.264	< 0.05*	1.3 (1.13–1.49)
ApaI			
Homo AA Aa and aa (r)	0.788	0.07	2.2 (0.9–5.1)
ApaI alleles			
A a (r)	0.744	0.022*	2.11 (1.11–3.97)

*OR* odds ratio

^*^P is significant if < 0.05

## Discussion

Vitiligo vulgaris was the most common clinical type of vitiligo in the present study (42%) and this is in agreement with many studies [21–24]. The current study showed that there was increased susceptibility of vitiligo in ApaI Aa genotype carriers as it was significantly more frequent in patients (48%) vs. controls (20%). Zhang et al. [6] conducted a meta-analysis about vitamin D receptor gene polymorphism, serum 25-hydroxyvitamin D levels and risk of vitiligo. For VDR ApaI polymorphism they identified 6 studies. They concluded that ApaI polymorphism is a potential biomarker for early detection of vitiligo. They found that the dominant genetic model (aa + Aa vs. AA), recessive genetic model (aa vs. Aa + AA), and allelic contrast model (a vs. A) of ApaI in the VDR gene were associated with a significant increased risk of vitiligo. In addition, Sajjad et al. [25] found that Aa genotype was significantly more frequent in Pakistani vitiligo patients (51%) than in controls (41%). In addition, Hassan et al. [7] reported that Aa genotype was significantly more frequent in Indian vitiligo patients (52%) vs. controls (27%).

Furthermore, Li et al. [26] in their meta-analysis about association of ApaI and BsmI polymorphisms with vitiligo risk demonstrated that, for the ApaI gene, the overall results of the included five studies showed no significant associations in any of the models. However, further subgroup analysis based on ethnicity showed a significant association under the dominant model (aa + Aa vs. AA) in the East Asian population, suggesting that East Asians who carry the ApaI a allele may be susceptible to vitiligo and there was no such association in Caucasian populations. They also concluded that ApaI genetic polymorphisms could be a potential biomarker for early detection of vitiligo.

The current study showed no significant difference in ApaI alleles distribution among patients and controls. Same results were reported by Aydingöz et al. [27] and Sajjad et al. [25]. On the other hand, the “a” allele was significantly more frequent in vitiligo patients than in controls in other studies [6, 7, 28, 29].

The current study demonstrated nonsignificant differences in TaqI genotypes and alleles distribution among vitiligo patients and controls. Zhang et al. [6], in their meta-analysis, identified four studies for VDR TaqI polymorphism in vitiligo and they observed a statistical association between VDR TaqI polymorphism and vitiligo susceptibility under the dominant genetic model (tt + Tt vs. TT). However, the data changed the pooled point estimate when converting the fixed effects model to the random effects model. No statistical association was observed under the recessive genetic model (tt vs. Tt + TT) and allelic contrast model (t vs. T). Therefore, TaqI may not be associated with an increased risk of vitiligo. This agrees with other studies [7, 25]. On the other hand, Aydingöz et al. [27] found that tt genotype and t allele were more frequent in Turkish vitiligo cases than in controls.

Finally, in comparison with another study on 75 Egyptian vitiligo patients and 75 age-matched healthy controls, Sobeih et al. [23] found in vitiligo patients that the frequency of the genotype variants aa of ApaI polymorphism and tt of TaqI polymorphism were both significantly higher in vitiligo patients than controls. On the other hand, the frequency of the genotype variant Tt was significantly lower in vitiligo patients than the controls. In addition, ApaI variant “a” allele frequency was significantly higher in vitiligo cases than controls, while TaqI variant t allele was not associated with susceptibility to vitiligo.

It may be normal to find differences in genotypic and allelic frequencies among patients in different studies due to different ethnic backgrounds in different countries, sample size differences, gender variability, differences in genetic backgrounds and working on other VDR gene polymorphisms [30].

In the current study, after following up vitiligo patients on NB-UVB phototherapy for 6 months, the response rate to NB-UVB was 66%. VASI score percent change showed significant decrease (45%) in responders with regimentation after 6 months (72 sessions) phototherapy. Yet, the non-responders showed more active disease with significantly higher VIDA score. This in agreement with other studies [15, 31]. In addition, Bae et al. [17] reported in their meta-analysis that at least mild response (≥ 25% repigmentation) occurred in 74.2% of 232 vitiligo patients in 11 studies after 6 months of treatment. However, Cabrera et al. [32] reported that the mean improvement was 45.7% after 72 sessions. Moreover, Silpa-Archa et al. [33] reported that nonsegmental vitiligo and segmental vitiligo demonstrated overall improvement in VASI score of –50.0% ± 31.0% and − 40.0% ± 28.3%, respectively, in patients received NB-UVB phototherapy twice to three times weekly for a median duration of 12 months.

The present study showed that there was no significant differences between responders and non-responders regarding age, sex, family history of vitiligo and presence of thyroid disease, while there was significant response in patients from urban areas. This agrees with many studies that stated that no association was found between response to phototherapy and sex, age, family history of vitiligo or presence of thyroid disease [15, 34, 35]. In addition, Anbar et al. [36] stated that no significant correlations were detected between the behavior of vitiligo during NB-UVB and any of the demographic or clinical data of the patients. However, another study showed that patients aged less than 20 years with recent vitiligo achieve more repigmentation [37].

The present study showed that mixed type of vitiligo was significantly higher in responders, while focal type was significantly higher in non-responders which agrees with many studies [32, 38, 39]. In addition, Silpa-Archa et al. [33] reported that factors predictive of good outcome of NB-UVB phototherapy included type of vitiligo, lesion location, disease duration before NB-UVB, and duration and total number of NB-UVB treatments.

In general, factors determining good response to NBUVB in vitiligo may be darker skin types, facial lesions, except for perioral lesions, lesions in non-acral areas, stable vitiligo lesions, patients who show early response (by 1st month), and treatment adherence [40–42].

Our study showed that the AA genotype was significantly higher in responders followed by Aa genotype, while all aa genotype carriers were non-responders. This coincides with our results of VIDA score of vitiligo activity. AA genotype carriers significantly had the least VIDA score, while Aa genotype carriers showed higher VIDA score and lastly the aa genotype carriers significantly showed the highest VIDA score. In addition, A allele distribution was significantly higher than a allele in responders. Furthermore, the current study found no significant association between TaqI genotypes or alleles and the response to NB-UVB therapy, VIDA score, VASI score or VASI score changes during treatment. Finally, the results of the present research represent some clinical and genetic predictors of good response to NB-UVB phototherapy in vitiligo as mixed type of vitiligo, urban residence, ApaI AA genotype and A allele, high VASI score before treatment, high percent of change in VASI score with treatment and low VIDA score.

To the best of our knowledge, this is the first study evaluated the association between the VDR gene polymorphisms (ApaI and TaqI) and the response of vitiligo to NB-UVB phototherapy. In psoriasis, there was a nearly similar study. Ryan et al. [43] studied clinical and genetic predictors of response to NB-UVB for the treatment of chronic plaque psoriasis. The frequencies of the Fok1, Apa1, Bsm1, Taq1 and rs4516035 polymorphisms of the VDR gene were assessed in 93 psoriasis patients. None of the VDR polymorphisms analysed was predictive of number of NB-UVB exposures to clearance.

In another study, cutaneous VDR expression and serum 25(OH) vitamin D level in vitiligo patients were significantly lower compared to controls. After NB-UVB therapy, there were significant rises in cutaneous VDR expression and serum 25(OH) vitamin D. VDR expression was significantly higher in repigmented skin compared to nonresponding lesion [11].

Although photocarcinogenic effect of NBUVB has not been proven [42], the British Association of Dermatologists recommended improved efficacy of treatment to reduce the amount of UV radiation exposure per treatment course [44]. In addition, as NB-UVB treatment is time consuming and expensive, the ability to predict patients who will clear quickly with prolonged remission would be useful in terms of patient care and health economics. While NB-UVB was shown to be a very effective treatment in most patients, a group of patients was detected who showed an inefficient treatment response [43].

Finally, there were some limitations of the current study as relatively small sample of the study population and lack of serum vitamin D estimation before and after phototherapy.

## Conclusions

This is the first prospective study to demonstrate that both clinical and genetic parameters may predict response to NB-UVB phototherapy in vitiligo patients. This study provides a paradigm for other studies, highlighting the need to evaluate both clinical and genetic factors when assessing treatment outcomes in vitiligo.

VDR gene polymorphisms may share in vitiligo pathogenesis and in vitiligo response to phototherapy as they may lead to VDR dysfunction and decreased vitamin D activity. However, routine testing for VDR polymorphisms is likely to be too expensive for use in clinical practice.

Further investigations for the role of different types of VDR gene polymorphisms in different types of vitiligo and in response to phototherapy are strongly recommended with larger sample size and longer durations of treatment and follow-up.
